# Time-Dependent Effects of Clinical Interventions on SARS-CoV-2 Immunity in Patients with Lung Cancer

**DOI:** 10.3390/vaccines12070713

**Published:** 2024-06-26

**Authors:** Philip C. Mack, Chih-Yuan Hsu, Ananda M. Rodilla, Jorge E. Gomez, Jazz Cagan, Yuanhui Huang, Sooyun Tavolacci, Rajesh M. Valanparambil, Nicholas Rohs, Rachel Brody, Brittney Nichols, Juan Manuel Carreño, Sheena Bhalla, Christian Rolfo, David E. Gerber, Amy Moore, Jennifer C. King, Rafi Ahmed, John D. Minna, Paul A. Bunn, Adolfo García-Sastre, Florian Krammer, Fred R. Hirsch, Yu Shyr

**Affiliations:** 1 Center for Thoracic Oncology, Tisch Cancer Institute, Icahn School of Medicine at Mount Sinai, New York, NY 10029, USA; ananda.rodilla@mssm.edu (A.M.R.); jorge.e.gomez@mssm.edu (J.E.G.); jazz.cagan@mssm.edu (J.C.); yuanhui.huang@mssm.edu (Y.H.); caroline.tavolacci@icahn.mssm.edu (S.T.); nicholas.rohs2@mountsinai.org (N.R.); christian.rolfo@mssm.edu (C.R.); fred.hirsch@mssm.edu (F.R.H.); 2 Department of Biostatistics, Vanderbilt University, Nashville, TN 37235, USA; chih-yuan.hsu@vumc.org (C.-Y.H.); yu.shyr@vumc.org (Y.S.); 3 Graduate School of Biomedical Sciences, Icahn School of Medicine at Mount Sinai, New York, NY 10029, USA; 4 Emory Vaccine Center, Emory University, Atlanta, GA 30322, USA; rajesh.murali.valanparambil@emory.edu (R.M.V.); rahmed@emory.edu (R.A.); 5 Department of Microbiology and Immunology, Emory University, Atlanta, GA 30322, USA; 6 Department of Pathology, Molecular and Cell Based Medicine, Icahn School of Medicine at Mount Sinai, New York, NY 10029, USA; rachel.brody@mountsinai.org (R.B.); adolfo.garcia-sastre@mssm.edu (A.G.-S.); florian.krammer@mssm.edu (F.K.); 7 GO2 Foundation for Lung Cancer, Washington, DC 20006, USA; bnichols@go2.org (B.N.); jking@go2.org (J.C.K.); 8 Department of Microbiology, Icahn School of Medicine at Mount Sinai, New York, NY 10029, USA; jm.carreno@mssm.edu; 9 Center for Vaccine Research and Pandemic Preparedness (C-VaRPP), Icahn School of Medicine at Mount Sinai, New York, NY 10029, USA; 10 Hamon Center for Therapeutic Oncology Research, Departments of Internal Medicine and Pharmacology UT Southwestern Medical Center, Dallas, TX 75235, USA; sheena.bhalla@utsouthwestern.edu (S.B.); david.gerber@utsouthwestern.edu (D.E.G.); john.minna@utsouthwestern.edu (J.D.M.); 11 LUNGevity Foundation, Bethesda, MD 20814, USA; amoore@lungevity.org; 12 Department of Internal Medicine, University of Colorado Cancer Center, Denver, CO 80045, USA; paul.bunn@cuanschutz.edu; 13 Department of Medicine, Division of Infectious Diseases, Icahn School of Medicine at Mount Sinai, New York, NY 10029, USA; 14 Global Health and Emerging Pathogens Institute, Icahn School of Medicine at Mount Sinai, New York, NY 10029, USA

**Keywords:** SARS-CoV-2 antibody levels, lung cancer therapies, time-dependent regression model

## Abstract

In patients with lung cancer (LC), understanding factors that impact the dynamics of severe acute respiratory syndrome coronavirus 2 (SARS-CoV-2) anti-spike antibody (SAb) titers over time is critical, but challenging, due to evolving treatments, infections, vaccinations, and health status. The objective was to develop a time-dependent regression model elucidating individual contributions of factors influencing SAb levels in LC patients using a prospective, longitudinal, multi-institutional cohort study initiated in January 2021. The study evaluated 296 LC patients—median age 69; 55% female; 50% stage IV. Blood samples were collected every three months to measure SAb levels using FDA-approved ELISA. Asymptomatic and unreported infections were documented through measurement of anti-nucleocapsid Ab levels (Meso Scale Discovery). Associations between clinical characteristics and titers were evaluated using a time-dependent linear regression model with a generalized estimating equation (GEE), considering time-independent variables (age, sex, ethnicity, smoking history, histology, and stage) and time-dependent variables (booster vaccinations, SARS-CoV-2 infections, cancer treatment, steroid use, and influenza vaccination). Significant time-dependent effects increasing titer levels were observed for prior SARS-CoV-2 infection (*p* < 0.001) and vaccination/boosters (*p* < 0.001). Steroid use (*p* = 0.043) and chemotherapy (*p* = 0.033) reduced titer levels. Influenza vaccination was associated with increased SAb levels (*p* < 0.001), independent of SARS-CoV-2 vaccine boosters. Prior smoking significantly decreased titers in females (*p* = 0.001). Age showed no association with titers. This GEE-based linear regression model unveiled the nuanced impact of multiple variables on patient anti-spike Ab levels over time. After controlling for the major influences of vaccine and SARS-CoV-2 infections, chemotherapy and steroid use were found to have negatively affected titers. Smoking in females significantly decreased titers. Surprisingly, influenza vaccinations were also significantly associated, likely indirectly, with improved SARS-CoV-2 titers.

## 1. Introduction

Prior to the availability of severe acute respiratory syndrome coronavirus 2 (SARS-CoV-2) vaccines, initial studies showed that patients with lung cancer (LC) were at increased risk of serious complications of SARS-CoV-2 infection, including hospitalization and death [1,2,3,4,5,6,7]. Increased mortality risk was associated with advanced age, male sex, smoking, number of comorbidities, performance status, and actively progressing cancer [8]. In another meta-analysis of cancer patients, factors significantly associated with mortality included age, male sex, hypertension, and diabetes, with LC having the highest case fatality rate (32.9%) among solid tumors [9].

Post-vaccination studies indicated that most LC patients developed durable antibody responses to SARS-CoV-2 vaccinations. However, multiple studies investigating SARS-CoV-2 spike protein antibody (SAb) titers, specifically in LC patients after vaccination, revealed a small subpopulation that appeared to have compromised immune responses. Our previous work revealed a small but significant percentage of patients (5%) with no detectable titer levels after full vaccination, a phenomenon not observed in healthy controls [10]. Valanparambil et al. found that 25% of LC patients showed notably poorer antibody responses (*p* < 0.001) [11]. Others have reported similar results, finding minimal to moderate deficits in SAb titers in LC patients but overall high (>90%) seroconversion rates [12,13].

The underlying clinical, demographic, and tumor biologic factors contributing to this suboptimal titer response in this vulnerable cancer population have not been fully elucidated. Potential reasons include the immunosuppressive activity of LC therapeutics and ancillary steroid use, the underlying malignancy, and history of cigarette smoking. When analyzing these effects, a substantial complicating factor stems from the timing and severity of SARS-CoV-2 infections and the timing of vaccinations and boosters relative to cancer-related events, including changes in disease status, cancer treatments, and use of steroids. We hypothesized that an analytic approach that takes into account the timing of vaccinations, SARS-CoV-2 infections, and cancer treatments relative to plasma draws conducted to measure Sab titers would provide more precise information regarding the effect of these events on immune response. To address this, we developed a time*-dependent* linear regression model to analyze the effects of cancer treatments, steroid use, demographics, infections, vaccinations, and other factors in the period immediately before anti-spike antibody titer level measurements. We applied this model to our large, well-annotated cohort of LC patients who have undergone serial plasma analysis every three months to measure Sab titer levels.

## 2. Materials and Methods

### 2.1. Study Design and Participant Data Collection

A prospective observational study (Mount Sinai STUDY-20-01470) received Institutional Review Board approval on 11 November 2020. This ongoing study is enrolling patients with LC treated at the Center for Thoracic Oncology, Tisch Cancer Institute at Mount Sinai Hospital, NY; the University of Texas Southwestern Medical Center in Dallas, TX; and National Jewish Health in Denver, CO. Adult patients diagnosed with LC of any stage, histology, or treatment status were eligible, regardless of SARS-CoV-2 infection or vaccination status. We continuously collected and updated clinical and demographic information, including previous SARS-CoV-2 infection, COVID-19 severity, vaccination (influenza and SARS-CoV-2) status and dates, patient self-reported smoking status, age at enrollment, co-morbidities, and treatments. Inclusion criteria were limited to an age of 18 years or older, no other ongoing cancer types, and ability to comprehend the informed consent form (produced in English, Spanish, Chinese, and Bengali). For this study, enrollment was limited to patients who had contributed at least one blood draw and were fully vaccinated, defined as two weeks after completion of initial vaccination series (for mRNA vaccines—after the second dose). Patients enrolled to the master study were not required to have received any vaccinations or boosters (SARS-CoV-2, viral influenza, or otherwise) or to have had documented SARS-CoV-2 infections; however, this sub-study analysis was limited to those who were fully vaccinated with the initial series. A comparison of these cohorts is shown in Table 1. Further cohort details are provided in our initial publication [10].

### 2.2. Blood Draws and Processing

Blood sample collection was planned at enrollment and every 3 months up to 24 months. For titer analyses, blood was drawn into ethylenediaminetetraacetic acid (EDTA) tubes using a double-spin approach, aliquoted, and stored at −80 °C.

### 2.3. SARS-CoV-2 Binding Antibody Assessment

Anti-spike antibodies to SARS-CoV-2 were measured using a well-established two step enzyme-linked immunosorbent assay (ELISA) [14,15,16,17], as previously reported for this cohort [10]. Anti-nucleocapsid antibody analysis was conducted in separately frozen aliquots using the methodology and dichotomizations previously reported [11,18].

### 2.4. Statistical Considerations

The primary objective of this longitudinal cohort study was to investigate the association of anti-spike antibody titers with clinical characteristics in patients with LC who have been fully vaccinated (defined as: two doses of mRNA vaccines, Moderna mRNA-1273 or Pfizer BNT162b2, or one dose of adenoviral vaccine, J&J Ad26.COV2.S). A time-dependent linear regression model with generalized estimating equations (GEE) [19] was applied (described in detail below), where the exchangeable correlation structure was used for the modeling of within-patient titers (logarithm base 10). We considered age at enrollment, self-reported gender, smoking history, race/ethnicity, cancer stage, and histology as time-*independent* variables, whereas time interval after being fully vaccinated, the use of anti-inflammatory medicine/anti-cancer treatments (steroids, chemotherapy, targeted therapy, or immunotherapy) within 30 days prior to the titer measurements, the receipt of influenza vaccine/SARS-CoV-2 booster within 90 days prior to the titer measurements, and the infection with SARS-CoV-2 prior to the titer measurements were considered time-*dependent* variables. In the primary regression model, the two continuous variables (age and time interval after being fully vaccinated) were transformed by cubic B-splines. Forest plots and waterfall plots were employed to visualize the significance and magnitude of association between the titers and the clinical characteristics. A sensitivity analysis was also conducted to assess the reliability of the findings (Supplementary Tables S4 and S5). In the sensitivity analysis, we excluded 16 titer measurements from 16 patients that occurred when both the influenza vaccine and the SARS-CoV-2 booster were administered within 90 days prior to the measurements and the two vaccines were administered within 10 days of each other. Furthermore, to explore the relationship between the titers and the clinical characteristics in different subgroups, we performed subgroup analyses, where the analysis cohort was divided by gender, ethnicity, or histology. All data analyses were carried out using base R 4.0.3 and the R packages Hmisc 4.5-0, mice 3.16.0, geepack 1.3-2, splines 4.0.3, and ggplot2 3.3.3.

### 2.5. Generalized Estimating Equations (GEE)

Let $Y_{ji}$ be the j-th measurement of log10(anti-S) after full vaccination of patient i, $j=1,\ldots ,n_{i}$ ($n_{i}$ varies with patients), and i=1,…,m (patients). Denote $\mu_{ji}$ and $\sigma_{ji}$ as the mean and the variance of $Y_{ji}$, respectively. We assume $\mu_{ji}=\beta^{T}x_{ji}$ and $\sigma_{ji}=\sigma$, where $x_{ji}={\left(1,\;x_{ji,1},\ldots ,x_{ji,p}\right)}^{T}$ is the vector of covariates, where covariates can be time-dependent or independent, and $\beta ={\left(\beta_{0},\beta_{1},\ldots ,\beta_{p}\right)}^{T}$ is the vector of coefficients to show the effect magnitudes. Also, we assume the correlation of $Y_{i}={\left(Y_{ji},j=1,\ldots ,n_{i}\right)}^{T}$ as $C_{i}\left(\rho \right)=\left(1-\rho \right)I_{i}+\rho J_{i}$, where $I_{i}$ is an identity matrix of dimension $n_{i}$, and $J_{i}$ is a square matrix where all elements are 1 and its dimension is the same as $I_{i}$. ρ is the intra-patient correlation.

With the measurements $Y_{i}$ and $x_{ji}$, $j=1,\ldots ,n_{i}$ and i=1,…,m, the coefficient estimate $\hat{\beta }$ for β can be obtained by solving the equation $\sum_{i=1}^{m}D_{i}^{T}V_{i}^{-1}(Y_{i}-\mu_{i})=0$, where $D_{i}=\partial \mu_{i}/\partial \beta^{T}$, $V_{i}=\sigma^{2}C_{i}\left(\rho \right)$, and $\mu_{i}={\left(\mu_{ji},j=1,\ldots ,n_{i}\right)}^{T}$. It is well-known from Ref. [20] that under mild regularity conditions, $\sqrt{m}(\hat{\beta }-\beta )$ approximates $N(0,mW^{-1}\sum_{i=1}^{m}D_{i}^{T}V_{i}^{-1}cov\left(Y_{i}\right)V_{i}^{-1}D_{i}W^{-1})$ when m is large, where W= $\sum_{i=1}^{m}D_{i}^{T}V_{i}^{-1}D_{i}$ and $cov\left(Y_{i}\right)$ is the covariance of $Y_{i}$. The estimate for $Var\left(\hat{\beta }\right)$ is given by $\hat{Var\left(\hat{\beta }\right)}={\hat{W}}^{-1}\sum_{i=1}^{m}{\hat{D}}_{i}^{T}{\hat{V}}_{i}^{-1}\hat{cov\left(Y_{i}\right)}{\hat{V}}_{i}^{-1}{\hat{D}}_{i}{\hat{W}}^{-1}$, where $\hat{cov\left(Y_{i}\right)}=\left(Y_{i}-{\hat{\mu }}_{i}\right){\left(Y_{i}-{\hat{\mu }}_{i}\right)}^{T}.$ With $\hat{\beta }$ and $\hat{Var\left(\hat{\beta }\right)}$, the confidence intervals and *p*-values can be calculated.

## 3. Results

### 3.1. Patients

Between 1 January 2021 and 13 March 2023, 398 patients were enrolled. For this analysis, 296 patients who had at least one SARS-CoV-2 antibody titer measurement and had received the full vaccination series at the time of data cut-off were included (Supplemental Figure S1). Of these, 267 were from the Tisch Cancer Institute at the Mount Sinai Hospital in New York City, 27 were from the University of Texas Southwestern in Dallas, and 2 patients were from National Jewish Health in Denver, Colorado. The median age was 69 (IQR: 62–76), 55% were female, and 26% self-reported as never-smokers. There were no significant differences between the enrolled cohort and the analysis cohort. Additional patient characteristics such as tumor stage, histology, first vaccination type, and cancer therapy are summarized in Table 1. The number and vaccination type sequence over time are shown by category and frequency in Supplemental Table S1.

### 3.2. Anti-Spike Antibody Titers over Time

**Figure 1:**
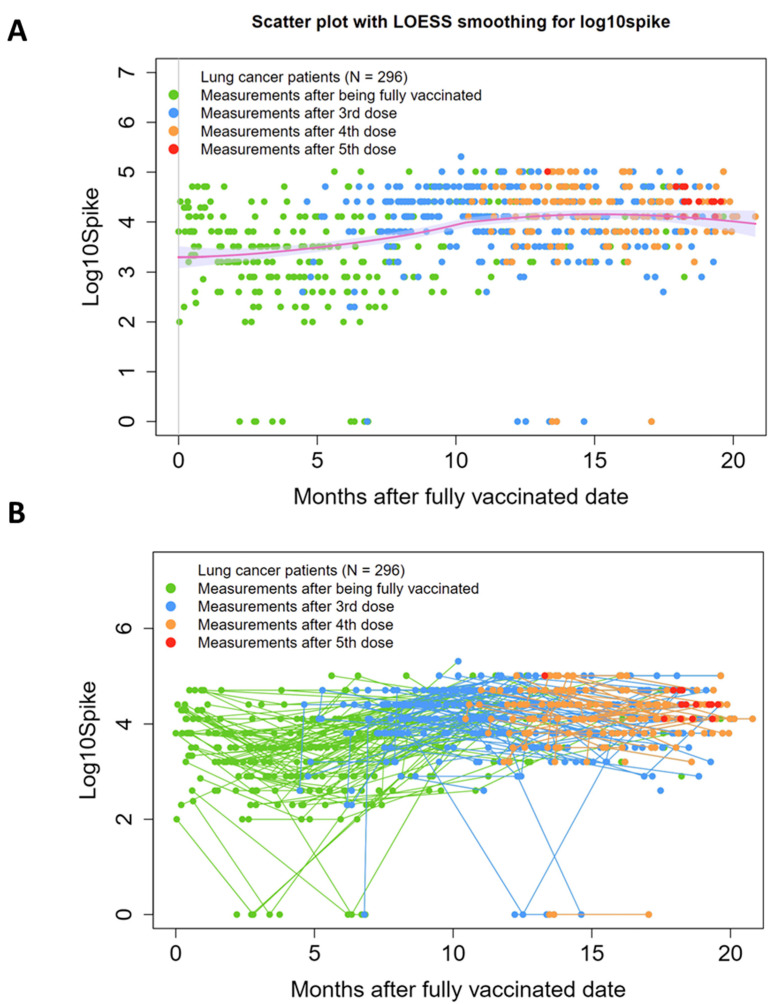
SARS-CoV-2 anti-Spike antibody titers (SAb) measurements over time. (**A**) Scatter plot with LOESS smoothing, with measurements shown as independent events with a trend line. (**B**) Spaghetti plot, with data from individual patients linked by a straight connecting line. Data points are color-coded based on number of vaccinations received, with “zero” on the x-axis representing time of full vaccination (two weeks past first dose of J&J or second dose of an mRNA-based vaccine). Y-axis: Log10 of Sab titers. Individual patients who received boosters trend toward increased titers over time.

SARS-CoV-2 anti-Spike antibody (SAb) measurements over time are shown in Figure 1A as independent events with a trend line. In Figure 1B, longitudinal SAb timepoints for each individual patient are connected by lines. Data points are color-coded based on number of vaccinations received, with “zero” on the x-axis representing time of full vaccination (two weeks past first dose of J&J or second dose of an mRNA-based vaccine). In this population, where 75% (223/296) of patients received at least one booster vaccination and 40% (119/296) received multiple boosters, the trend line shows an upward trend approximately 1 year after initial vaccination.

As we reported previously [10], there was a small contingent of patients (n = 11, 5%) that showed at least one SAb reading of zero after full vaccination. In seven of these cases, subsequent booster vaccinations resulted in seroconversion to positive readings, with the exception of one patient, who maintained a zero titer through two boosters. Nucleocapsid antibody titers (anti-N) were also modest in these patients, with three achieving positivity by our pre-established cut-off [18]. Anti-S and anti-N levels over time for patients with at least one zero SAb reading after full vaccination are shown in Supplemental Figure S2A,B, respectively.

### 3.3. Model Findings

**Figure 2:**
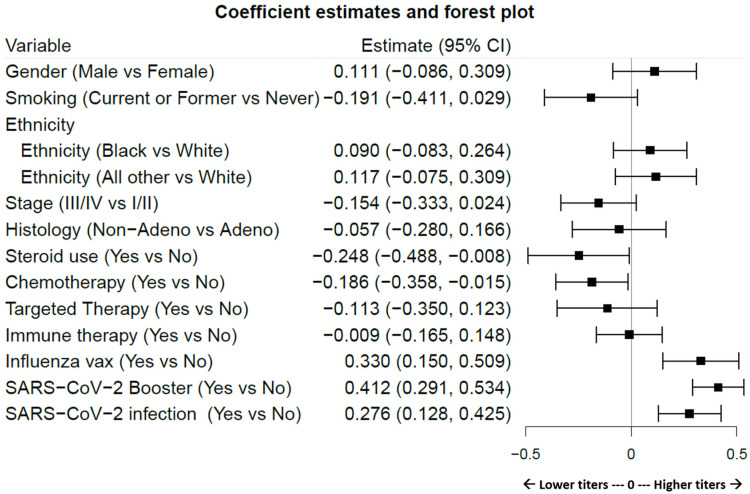
Forest plot visualization of the magnitude with confidence intervals showing the effect of each variable on Sab titers. Estimates to the left of the zero line indicate decreasing effects on titer levels and to the right show increasing effects. Variable definitions and limits are articulated in Table 2. Table 3 shows numeric values. Significantly increased effects are observed subsequent to SARS-CoV-2 boosters, SARS-CoV-2 infections, and influenza vaccination. Significantly decreased effects are observed after chemotherapy and steroid use.

Variable considerations are presented in Table 2, with time *dependence* or time *independence* indicated. Variables that apply to all patients (age, gender, smoking history, ethnicity, cancer stage, histology, and time after full vaccination) are scored for completeness of data. Variable events which include specific cancer-related treatment interventions (steroid use, cancer therapies categorized as chemotherapy, targeted therapy, and immunotherapy), SARS-CoV-2 and influenza vaccinations, and SARS-CoV-2 infection are annotated for proportion of patients affected. SARS-CoV-2 infection rates were calculated as a product of clinically documented infections and/or nucleocapsid Ab positivity using previously described definitions [18]. Figure 2 displays the effects of antibody titers according to patient, cancer, and SARS-CoV-2 characteristics. The effect magnitude, p-value, and confidence intervals are shown in Table 3, with nonlinear effects of time after full vaccination and age detailed in Supplemental Table S3. In this cohort, steroid use (*p* = 0.043) and chemotherapy (*p* = 0.033), but not targeted or immune therapy, were significant contributors to decreased titers, whereas SARS-CoV-2 vaccination/boosters (*p* < 0.001), SARS-CoV-2 infection (*p* < 0.001), and surprisingly, influenza vaccination (*p* < 0.001) were independent significant contributors to increased titer levels. A history of tobacco smoking trended towards a decrease in SAb levels, but did not achieve significance in the general population (*p* = 0.089). Age in this LC population did not appear to influence titer levels (*p* = 0.819, a Chi-square test of df = 3 for the three components of age). In addition, *time after full vaccination* was associated with a population-level net increase in titer levels (*p* < 0.001, a Chi-square test of df = 3 for the three components of the time variable), peaking approximately sixteen months after full vaccination before trending downward.

**Figure 3:**
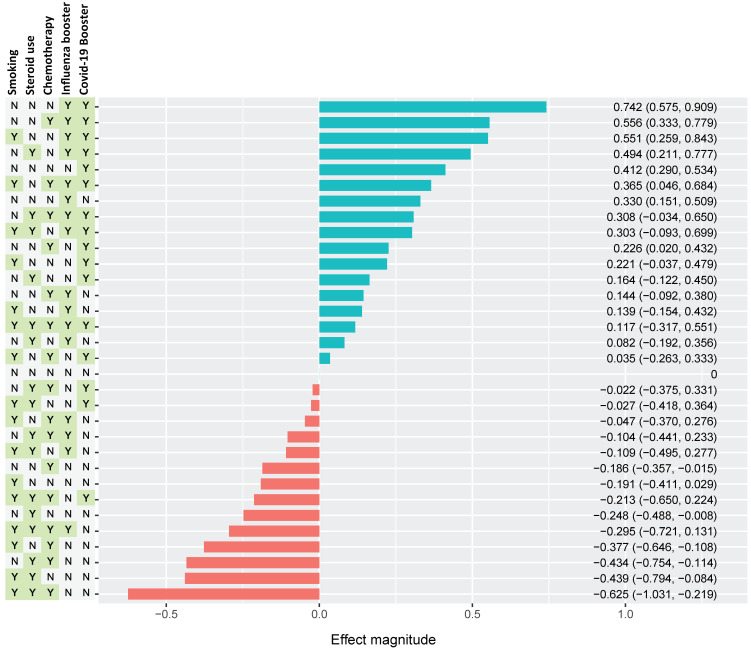
Estimated combined effect of multiple variables on Sab titers. Selected variables include smoking history, steroid use, chemo use, influenza vaccination, and SARS-CoV-2 vaccination as yes (Y) or no (N). Effect magnitude is indicated as positive (teal) or negative (red) annotated for magnitude estimate and CIs. The variable groups are ordered vertically, with the most positive combination at the top and the most negative combination at the bottom (normalized to an “all zero” configuration). The most positive effect was observed in the N-N-N-Y-Y, which indicates a grouping of never-smokers with no recent steroid or chemo use, as well as recent receipt of both influenza and SARS-CoV-2 vaccines. The most negative effect occurs in group Y-Y-Y-N-N, with positive smoking history with recent use of both steroids and chemotherapy, but no recent receipt of vaccine boosters.

The combined effects of multiple factors on SAb levels are estimated as a waterfall plot in Figure 3. Here, the individual and combined effects of smoking, steroid use, chemotherapy use, influenza vaccine, and SARS-CoV-2 vaccination/booster are shown in every possible combination, as indicated by the presence (Y) or absence (N) in the left columns. The waterfall plot is organized from most positive collective effect to most negative effect. The greatest positive effect on SAb levels resulted from a combination of recent SARS-CoV-2 booster vaccination, recent influenza vaccination, and an absence of smoking history, steroid use, and chemotherapy use. The greatest negative effect was observed in patients with a combined history of smoking, steroid use, receipt of recent chemotherapy, and temporal distance from vaccinations.

### 3.4. Differences by Gender

The distribution of analysis variables between females (n = 164) and males (n = 132) is shown in Supplemental Table S2. Females were more likely to be never-smokers (32% vs. 18%; *p* = 0.007) and more likely to be diagnosed with adenocarcinoma histology (74% vs. 60%; *p* = 0.008). Tumor stage and ethnicity did not significantly differ by gender. The effect estimate by forest plot is shown in Supplemental Figure S3 for each gender. In females, a significant negative effect from smoking on SAb levels was observed, which was not seen in males (who had an overall significantly increased smoking history). In contrast, the positive effect on SAb levels from influenza vaccination trended in females, but was significant in males. In males, the vaccination effect (both SARS-CoV-2 and influenza), along with SARS-CoV-2 infection, were more pronounced.

## 4. Discussion

As COVID-19 segues into an endemic problem, strategies now turn to identifying vulnerable populations, including cancer patients, that may respond suboptimally to vaccination and/or are at greater risk of severe COVID-related disease. Aspects of SARS-CoV-2 that make it particularly dangerous are its ability to evolve and rapidly proliferate new variants, as exemplified by its penchant for zoonotic infections [20]. With increased survival rates in advanced non-small cell LC patients due to modern therapies, ongoing studies necessarily evolved along with the pandemic to investigate potential interactions of these two diseases and implications for cancer therapies. However, such longitudinal studies are challenging due to the continuing, patient-specific evolution of both diseases. The principal obstacle stems from the highly variable timing of key events that serve as critical variables when studying the SARS-CoV-2—lung cancer juxtaposition. From the cancer care perspective, therapy, disease status, steroid use, and secondary interventions are constantly evolving. From the COVID-19 side, booster vaccinations, infections with emerging variants, and specific infection therapies occur sporadically. These time-*dependent* variables complicate the interpretation of SARS-CoV-2 antibody titer levels (vaccine- or infection-induced) already influenced by time-*independent* variables (ethnicity, age at diagnosis, gender, smoking history) and disease-specific variables (stage, histology).

To directly address this challenge, we developed a time-dependent linear regression model incorporating an estimating equation using an exchangeable correlation structure for modeling the association of within-participant titers. The model proved necessary in order to disentangle the effects of time-dependent variables on longitudinal SAb levels. This analysis revealed several key effects that were not previously discernable without an understanding of the events and interventions immediately preceding each titer reading. Obvious time-based effects on titer levels included booster shots and infections, both of which proved, as expected, to have a significant impact on SAb levels. By accounting for these large effects in a temporal context, we were able to explore the more subtle impact of other events and interventions.

One such feature was the negative effect of chemotherapy on titer levels. Modeling revealed a significant decline in patient SAb levels after chemotherapy treatment, which were not observed after targeted therapy or immunotherapy. In many cases, patients may have received combined treatment with chemotherapy and immune therapy; however, only chemotherapy had an impact on titer levels. The model assessed chemotherapy impact, if it was received within a 30-day window prior to titer measurement. Previous LC studies have been mixed in terms of identifying an effect of chemotherapy on titer levels. Poor seroconversion was linked to various cancer therapies in solid tumors, of which 10% were LC [21]. Others did not observe an effect in specific LC populations [11,12,22]. Bowes et al. compared LC patients receiving radiotherapy to two separate control groups, showing significantly lower SAb levels in the treated group, although this group also had other concurrent immune suppressive conditions [23]. In our study, radiotherapy was not investigated.

Steroid use also emerged as a significant negative factor in regards to titer levels when evaluated in proximity to titer readings. In this population, steroids were sporadically used in response to acute conditions such as COPD exacerbation, radiation pneumonitis, or checkpoint inhibitor toxicity. Its modest but significant impact was unseen when using a static, population-based model. While we expected to see that steroid use could have an adverse effect on titer levels, its influence only became statistically apparent when using time-adjacent measurements, an indicator of this model’s effectiveness.

The effect of prior smoking was observed specifically and most strongly in females. Whether this was causative or associative with the significantly increased number of female never-smoker patients in this study, and the consequently significantly higher rate of adenocarcinoma, cannot be determined. Trontzas et al. observed an association between active smokers and lower post-vaccination anti-SARs-CoV-2 spike titer levels, but with no comparison of gender [24].

An intriguing observation from this study was the effect of influenza vaccines on SARS-CoV-2 titer levels. This unexpected finding appears to be independent of whether the patients received this vaccination within the same time window with a SARS-CoV-2 booster. However, a sufficient number of patients also received those vaccinations at different times or received just one or the other, allowing for the effects to be measured independently. In the overall population and in the male-only subgroup, receipt of an influenza vaccination within 90 days prior to a titer reading had a significant positive effect on SAb levels. The underlying mechanism for this effect is currently being explored, and it certainly cannot be ruled out that it is associative rather than causative. For instance, patients in better health or with a better performance status may opt for receiving the influenza vaccination in addition to SARS-CoV-2 vaccine, creating an untestable bias. Future studies of cancer vaccines may consider inclusion of patient vaccination and infection history.

While the effects of SARS-CoV-2 infection and booster vaccinations are expected to increase SAb levels, as confirmed in this dataset, a modeling strategy that accounts for these large effects was nevertheless essential to investigate more subtle effects of other variables. To identify mild or asymptomatic infections, anti-SARS-CoV-2 nucleocapsid antibody levels were measured as a proxy for infection to supplement reported incidents. This analysis revealed a substantial and significant increase in infection rates for this study population compared to the documented infection rate alone, as described recently [18]. In this fully vaccinated population, we did not observe many severe complications from COVID-19, as was the case in patients prior to vaccination.

At the population level, the positive influences on titer levels from vaccination boosters and infections outweighed the negative effects from variables such as smoking history, chemotherapy, and steroid use, as well as the expected generalized decline of titer levels over time. This resulted in a net increase in population SAb levels for the first year or so before the levels plateaued and ultimately diminished approximately 16 months after full vaccination. Despite a subset of patients who initially did not respond to vaccination [10], median anti-spike titer levels in this fully vaccinated LC population remained elevated more than 20 months after vaccination.

Factors that did not appear to significantly affect SAb levels in our population of patients with LC included age, tumor stage, histology, ethnicity, and gender. In the current study, the median age was 69, and a lack of representation from younger individuals (typical to LC cohorts) may skew the results. Comparisons between tumor stages showed non-significant trends towards higher stages associated with lower SAb levels, but significance was not observed in the general population (comparison of Stage I/II versus III/IV: *p* = 0.090), but trended in the male-only subgroup (*p* = 0.054). Patients of Caucasian descent represented 47% of the analysis dataset, with African Americans representing 20%, but no significant distinctions were observed in terms of variable effects on SAb levels between race/ethnicity (*p* = 0.407).

Beyond these clinically important immediate concerns involving vaccine-induced protection against SARS-CoV-2 infection, there is a significant knowledge gap regarding factors that influence B and T cell immunologic responses in LC patients in general. With the advent of mRNA vaccines directed against tumor acquired neo-antigens [25], it is important to develop statistical approaches, such as the model developed and used here, as well as baseline data to help guide these studies. It will be of great interest to see if these anti-SARS-CoV-2 results are mirrored by those for anti-neoepitope vaccines, illustrating the need to prospectively plan for the long-term serial collection of multi-factorial data in such studies.

## 5. Conclusions

Advanced modeling that takes into account the timing of key events relative to longitudinal measurements of SARS-CoV-2 antibody levels provides a more detailed and granular assessment of variables that can positively or negatively impact serology. These studies revealed the expected positive effects of SARS-CoV-2 infection and booster vaccinations on Sab titer levels, as well as the negative effects of steroid use and chemotherapy treatment. Additionally, an unanticipated positive impact of influenza vaccination was observed on patient titers. Future studies, both specifically in the context of COVID-19 and LC, as well as for all types of other health studies that rely on the long-term serial collection of multi-factorial data, could benefit from similar modeling.

## Figures and Tables

**Table 1:** Demographics.

	Full Cohort	Analysis Cohort
	N = 398	N = 296
**Gender**		
Female	53% (209)	55% (164)
Male	44% (175)	45% (132)
Missing	4% (14)	0% (0)
**Ethnicity**		
Asian	8% (30)	8% (25)
Black/African American	20% (79)	20% (59)
Hispanic	9% (36)	9% (28)
Hawaiian/Pacific islander	0% (1)	0% (1)
White	44% (176)	47% (138)
Missing	19% (76)	15% (45)
**Smoking**		
Never	23% (93)	26% (77)
Current or Former	71% (281)	74% (218)
NA	6% (24)	0% (1)
**Stage**		
1	10% (39)	10% (30)
2	7% (28)	8% (24)
3	25% (98)	27% (79)
4	46% (183)	50% (148)
Missing	13% (50)	5% (15)
**Histology**		
Adenocarcinoma	64% (253)	68% (201)
Large cell	1% (3)	1% (3)
Mixed	1% (2)	0% (1)
NSCLC NOS	6% (23)	7% (21)
Other	1% (5)	2% (5)
Small cell	8% (31)	6% (18)
Squamous Carcinoma	15% (58)	16% (46)
Missing	6% (23)	0% (1)
**First vaccine type**		
Johnson	4% (15)	4% (12)
Moderna	29% (115)	33% (97)
Moderna BA.4/BA.5	0% (1)	1% (1)
Pfizer	56% (221)	63% (187)
Missing	12% (46)	0% (0)
**Age**		
Med (IQR)	68 (61–68)	69 (62–76)

**Table 2:** Variables Considered in Model.

Variable	Time-Dependency	Completeness	Notes
Age	Independent	100%	Age at time of enrollment
Sex	Independent	100%	
Smoking history	Independent	99.7%	Never vs current/former
Ethnicity	Independent	85%	
Cancer stage	Independent	95%	At time of enrollment
Histology	Independent	99.7%	SCLC and NSCLC sub-histologies
Time after full vaccination	Dependent	100%	The time interval after the patient received a full vaccination
**Variable Event**		**Percent (N)**	
Steroid use	Dependent; 30 Days	32.4%	Systemic steroids such as prednisone, methylprednisolone, dexamethasone, hydrocortisone, or other, used long-term (>7 days).
Chemotherapy	Dependent; 30 Days	64.5%	Taxanes, platinum, pemetrexed, gemcitabine, vinorelbine, or other
Targeted Therapy	Dependent; 30 Days	18.6%	Osimertinib, erlotinib, gefitinib, afatinib, dacomitinib, bevacizumab, ramucirumab, crizotinib, alectinib, brigatinib, ceritinib, lorlatinib, entrectinib, selpercatinib, capmatinib, pralsetinib, larotrectinib, cabozantinib, vandetanib, tepotinib, or other
Immune therapy	Dependent; 30 Days	64.5%	Pembrolizumab, nivolumab, atezolizumab, durvalumab, cemiplimab, ipilimumab, or other
SARS-CoV-2 Booster	Dependent; 90 Days	77%	See Supplementary Table S3
Influenza vaccination	Dependent; 90 Days	100%	Unless administered concurrently with SARS-CoV-2 booster
SARS-CoV-2 infection	Dependent; prior to reading	51.7%	Includes any report of a positive test or anti-N plasma values above the 2000 unit threshold prior to the titer measurement.

“Completeness” indicates the completeness of data for variables that apply to all patients. “Days” indicates the amount of time prior to a blood reading in which the event was considered. Variable Events indicates an optional intervention or a SARS-CoV-2 infection.

**Table 3:** Impact of Variables.

Variable	Effect Magnitude	*p*-Value	Confidence Interval
Sex *	0.111	0.270	−0.086 to 0.309
Smoking history	−0.191	0.089	−4.110 to 0.029
Ethnicity (Black vs. White)	0.090	0.308	−0.830 to 0.264
Ethnicity (All other vs. White)	0.117	0.233	0.075 to 0.309
Cancer stage (III/IV vs. I/II)	−0.154	0.090	−0.333 to 0.024
Histology (adeno vs. non)	−0.057	0.619	−0.280 to 0.166
Steroid use	−0.248	**0.043**	−0.488 to −0.008
Chemotherapy	−0.186	**0.033**	−0.358 to −0.015
Targeted Therapy	−0.113	0.347	−0.350 to 0.123
Immune therapy	−0.009	0.912	−0.165 to 0.148
Influenza vax	0.330	**<0.001**	0.150 to 0.509
SARS-CoV-2 booster	0.412	**<0.001**	0.291 to 0.534
SARS-CoV-2 infection	0.276	**<0.001**	0.128 to 0.425

Negative effects (those associated with decreased titers) are indicated in red. Significant findings are **bolded**. * Nonlinear effects of “Time after full vaccination” and “Age” on titers are shown in Supplemental Table S3.

## Data Availability

Access to data generated by this study may be requested in accordance with SeroNET and Mount Sinai policies.

## Supplementary Materials

The following supporting information can be downloaded at: https://www.mdpi.com/article/10.3390/vaccines12070713/s1, Figure S1: Consort plot; Figure S2: “zero “ titer patients. A. Spaghetti plot showing longitudinal SAb levels in the subset of patients who had at least one “zero” titer reading after full vaccination (n = 11). B. The same patient set with graph showing anti-nucleocapsid Ab titers.; Figure S3: Forest plots with estimated combined effect of multiple variables on SAb levels dichotomized by sex. Table S1. Patient-level differences in the sequence of administration of vaccinations by type in the entire cohort. Table S2. Population-level differences between females and males in the analysis cohort (n = 296). Statistical tests used: Fisher’s exact test for discrete data; Wilcoxon test for continuous data. Table S3. Effects of Time after fully vaccinated and Age in main analysis. Table S4. Sensitivity analysis. Table S5. Effects of Time after fully vaccinated and Age in sensitivity analysis.
