# Supplementary material for: Time-Dependent Effects of Clinical Interventions on SARS-CoV-2 Immunity in Patients with Lung Cancer
Source: Vaccines (Basel). 2024 Jun 26;12(7):713. doi: 10.3390/vaccines12070713 (PMC11281667; doi:10.3390/vaccines12070713)
Supplement: Supplementary file 1 [file vaccines-12-00713-s001.zip › vaccines-3015603-supplementary.pdf]

Supplemental Figure S1. Consort plot

Frequencies of Site N=296

Site	Counts	% of Total
Mount Sinai	267	90.2 %
National Jewish Health	2	0.7 %
UT Southwestern	27	9.1 %

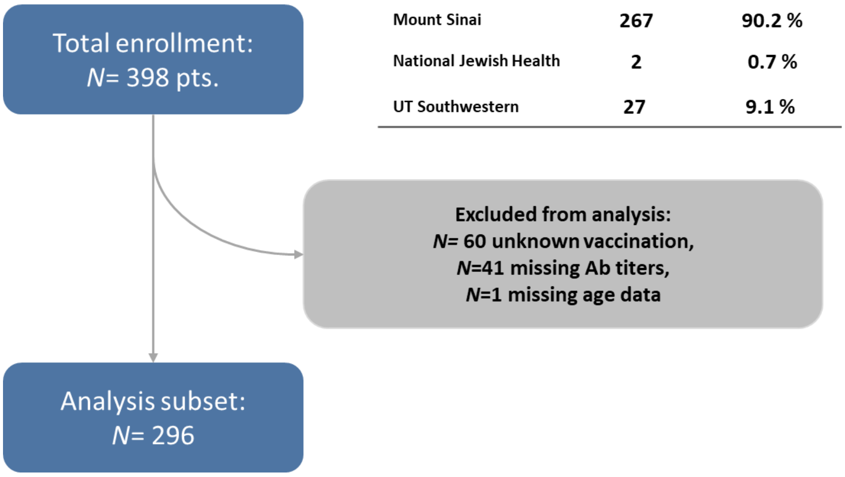

Supplementary Figure S1. Consort plot.

Supplemental Figure S2.

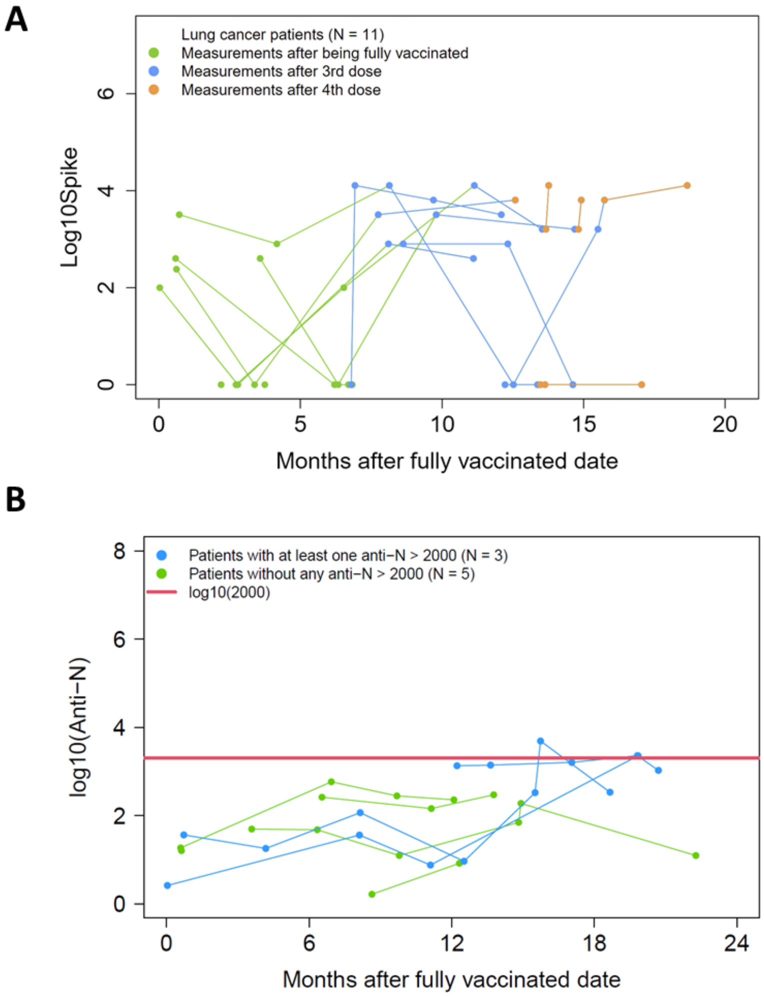

Supplementary Figure S2. "zero " titer patients. **A.** Spaghetti plot showing longitudinal SAb levels in the subset of patients who had at least one "zero"

titer reading after full vaccination (n = 11). **B.** The same patient set with graph showing anti-nucleocapsid Ab titers.

Supplemental Figure S3.

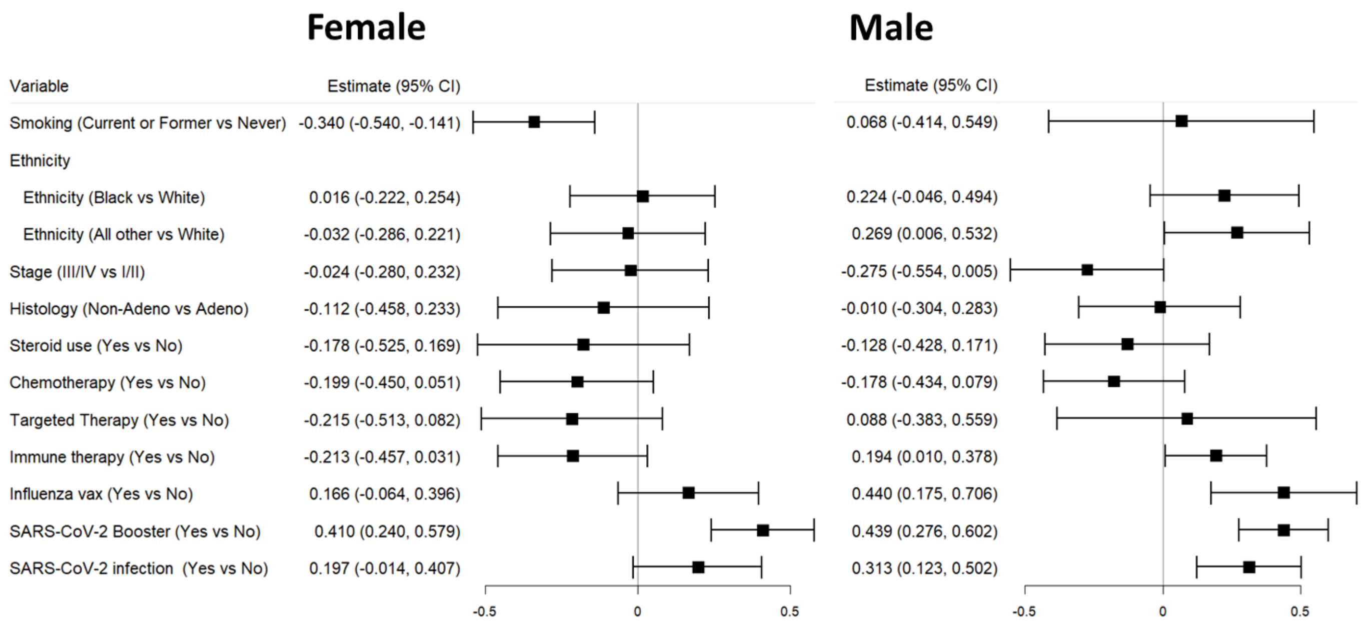

Supplementary Figure S3. Forest plots with estimated combined effect of multiple variables on SAb levels dichotomized by sex.

Supplemental Table S1. Vaccine type and Sequence (n =398)

Supplementary Table S1. Patient-level differences in the sequence of administration of vaccinations by type in the entire cohort.

1st shot	2nd shot	3rd shot	4th shot	5th shot	% (*N*)
Johnson	Johnson	Moderna	none	none	0% (1)
Johnson	Moderna	Moderna	Moderna BA.4/BA.5	none	0% (1)
Johnson	Moderna	none	none	none	1% (2)
Johnson	Moderna	Pfizer	Pfizer BA.4/BA.5	none	0% (1)
Johnson	none	none	none	none	2% (7)
Johnson	Pfizer	none	none	none	1% (3)
Moderna	Moderna	Moderna	Moderna	Moderna	1% (2)
Moderna	Moderna	Moderna	Moderna	Moderna BA.4/BA.5	3% (11)
Moderna	Moderna	Moderna	Moderna	none	4% (16)
Moderna	Moderna	Moderna	Moderna	Pfizer BA.4/BA.5	1% (3)
Moderna	Moderna	Moderna	Moderna BA.4/BA.5	Moderna BA.4/BA.5	0% (1)
Moderna	Moderna	Moderna	Moderna BA.4/BA.5	none	1% (4)
Moderna	Moderna	Moderna	none	none	9% (37)
Moderna	Moderna	Moderna	Pfizer	none	1% (4)
Moderna	Moderna	Moderna	Pfizer	Pfizer BA.4/BA.5	1% (2)
Moderna	Moderna	Moderna	Pfizer BA.4/BA.5	none	0% (1)
Moderna	Moderna	Moderna BA.4/BA.5	none	none	0% (1)
Moderna	Moderna	none	none	none	6% (23)
Moderna	Moderna	Pfizer	Moderna	none	0% (1)
Moderna	Moderna	Pfizer	none	none	1% (5)
Moderna	Moderna	Pfizer	Pfizer	none	1% (3)
Moderna	Moderna	Pfizer	Pfizer BA.4/BA.5	none	0% (1)
Moderna BA.4/BA.5	none	none	none	none	0% (1)
Pfizer	none	none	none	none	3% (10)
Pfizer	Pfizer	Johnson	Pfizer	none	0% (1)
Pfizer	Pfizer	Moderna	Moderna	Pfizer BA.4/BA.5	0% (1)
Pfizer	Pfizer	Moderna	none	none	1% (3)
Pfizer	Pfizer	Moderna	Pfizer BA.4/BA.5	none	0% (1)
Pfizer	Pfizer	Moderna BA.4/BA.5	Pfizer BA.4/BA.5	none	0% (1)
Pfizer	Pfizer	none	none	none	13% (52)
Pfizer	Pfizer	NA	Pfizer	none	0% (1)
Pfizer	Pfizer	Pfizer	Moderna	none	1% (2)
Pfizer	Pfizer	Pfizer	none	none	19% (74)
Pfizer	Pfizer	Pfizer	Pfizer	Moderna BA.4/BA.5	1% (5)
Pfizer	Pfizer	Pfizer	Pfizer	none	6% (22)
Pfizer	Pfizer	Pfizer	Pfizer	Pfizer	1% (4)
Pfizer	Pfizer	Pfizer	Pfizer	Pfizer BA.4/BA.5	8% (32)
Pfizer	Pfizer	Pfizer	Pfizer BA.4/BA.5	none	2% (9)
Pfizer	Pfizer	Pfizer	Pfizer BA.4/BA.5	Pfizer BA.4/BA.5	0% (1)
Pfizer	Pfizer	Pfizer BA.4/BA.5	none	none	1% (2)
NA	none	none	none	none	0% (1)
none	none	none	none	none	11% (45)

With initial vaccination series represented by the first one (single dose for Johnson) or two (two doses for Moderna and Pfizer) entries followed by boosters. The number in parentheses indicates the number of patients that correspond to that pattern of administration. “none” denotes no record of vaccination or booster in the indicated period. “NA” denotes brand and type missing/not reported, but patient known to have received an injection. “Johnson” = Ad26.COV2.S; “Moderna” = Original monovalent mRNA-1273; “Pfizer” = Original monovalent BNT162b2; “Moderna BA.4/BA.5” = mRNA-1273.222 (Bivalent Original/Omicron BA.4/BA.5); “Pfizer BA.4/BA.5” = BNT162b2 BA.4/BA.5 bivalent.

Supplementary Table S2. Population-level differences between females and males in the analysis cohort (n = 296). Statistical tests used: Fisher's exact test for discrete data; Wilcoxon test for continuous data.

	Female (N=164)	Male (N=132)	Combined (N=296)	Test Statistic
**Age, median (IQR)**	69 (62-77)	69 (62-75)	69 (62-76)	P=0.624
**Smoking**				
Never	32% (53)	18% (24)	26% (77)	P=0.007
Current or Former	67% (110)	82% (108)	74% (218)	
Missing	1% (1)	0% (0)	0% (1)	
**Cancer stage**				
1	13% (21)	7% (9)	10% (30)	P=0.139
2	10% (16)	6% (8)	8% (24)	
3	22% (36)	33% (43)	27% (79)	
4	51% (83)	49% (65)	50% (148)	
Missing	5% (8)	5% (7)	5% (15)	
**Ethnicity**				
White	45% (74)	48% (64)	47% (138)	P=0.150
Black/African American	24% (40)	14% (19)	20% (59)	
Others	18% (29)	19% (25)	18% (54)	
Missing	13% (21)	18% (24)	15% (45)	
**Histology**				
Adenocarcinoma	74% (122)	60% (79)	68% (201)	P=0.008
Non-Adenocarcinoma	25% (41)	40% (53)	32% (94)	
Missing	1% (1)	0% (0)	0% (1)	

Supplementary Table S3. Effects of *Time after fully vaccinated* and *Age* in main analysis.

Variable	Effect magnitude	p-value	Confidence Interval
**Time after fully vaccinated**			
Basis(Time after fully vaccinated) 1 [a]	-1.948	<0.001	-2.607 to -1.289
Basis(Time after fully vaccinated) 2 [a]	2.050	<0.001	1.496 to 2.605
Basis(Time after fully vaccinated) 3 [a]	-0.574	0.001	-0.914 to -0.233
**Age**			
Basis(Age) 1 [b]	-0.322	0.513	-1.286 to 0.642
Basis(Age) 2 [b]	-0.151	0.725	-0.994 to 0.692
Basis(Age) 3 [b]	-0.053	0.882	-0.757 to 0.650

[a] Three cubic B-spline basis functions that were used to constitute a non-linear transform for *Time after fully vaccinated.*

[b] Three cubic B-spline basis functions that were used to constitute a non-linear transform for *Age.*

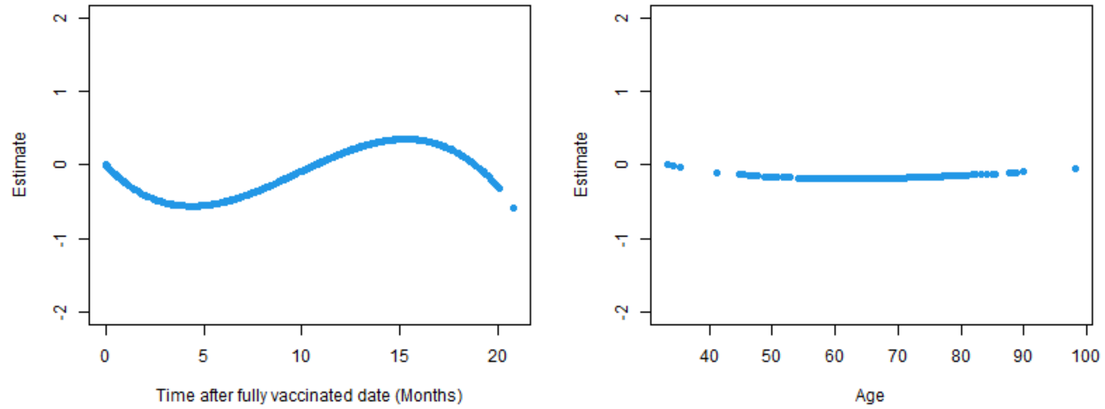

Nonlinear effect of *Time after fully vaccinated* (left) and *Age* (Right) for anti-spike antibody titer.

Supplementary Table S4. Sensitivity analysis.

Variable	Effect magnitude	p-value	Confidence Interval
Sex	0.110	0.281	-0.090 to 0.311
Smoking history	-0.204	0.076	-0.430 to 0.021
Ethnicity (Black vs White)	0.086	0.335	-0.089 to 0.261
Ethnicity (All other vs White)	0.115	0.247	-0.079 to 0.309
Cancer stage (III/IV vs I/II)	-0.139	0.131	-0.319 to 0.041
Histology (adeno vs non)	-0.054	0.637	-0.277 to 0.169
Steroid use	-0.250	0.047	-0.497 to -0.003
Chemotherapy	-0.186	0.036	-0.360 to -0.012
Targeted Therapy	-0.100	0.399	-0.332 to 0.132
Immune therapy	-0.006	0.938	-0.160 to 0.148
Influenza vax	0.364	<0.001	0.163 to 0.564
SARS-CoV-2 Booster	0.418	<0.001	0.297 to 0.540
SARS-CoV-2 infection	0.300	<0.001	0.147 to 0.452

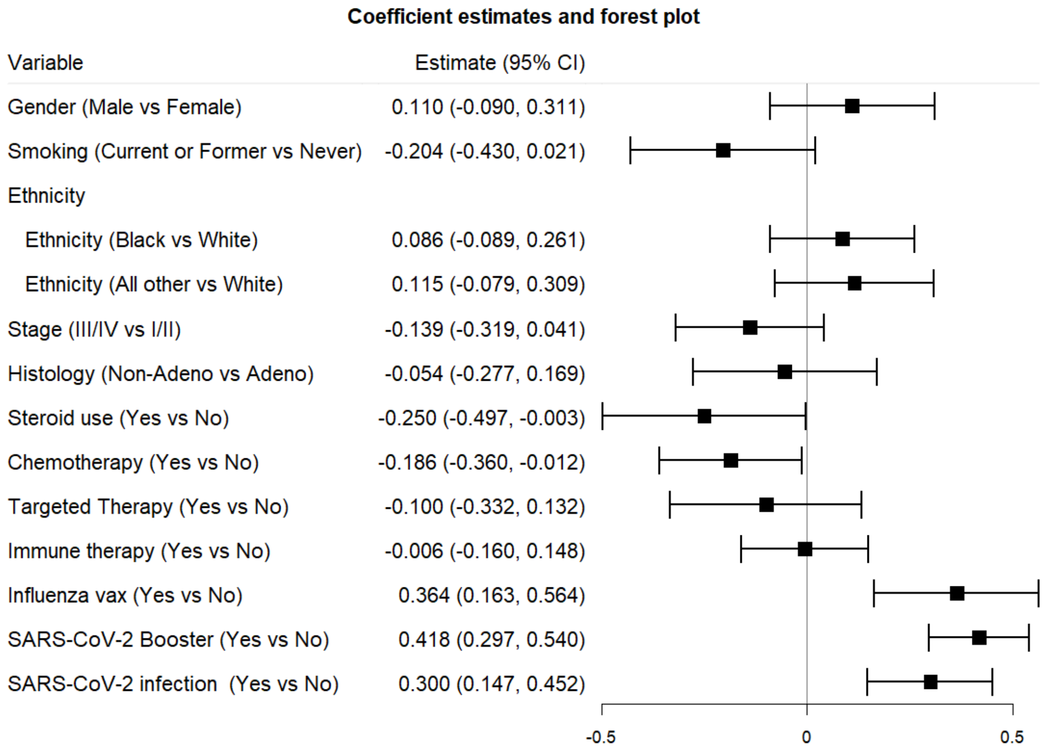

Supplementary Table S5. Effects of *Time after fully vaccinated* and *Age* in sensitivity analysis.

Variable	Effect magnitude	p-value	Confidence Interval
**Time after fully vaccinated**			
Basis(Time after fully vaccinated) 1 [a]	-1.981	<0.001	-2.646 to -1.315
Basis(Time after fully vaccinated) 2 [a]	2.014	<0.001	1.454 to 2.575
Basis(Time after fully vaccinated) 3 [a]	-0.557	0.002	-0.901 to -0.212
**Age**			
Basis(Age) 1 [b]	-0.493	0.309	-1.443 to 0.457
Basis(Age) 2 [b]	-0.173	0.692	-1.031 to 0.684
Basis(Age) 3 [b]	-0.161	0.650	-0.855 to 0.534

[a] Three cubic B-spline basis functions that were used to constitute a non-linear transform for *Time after fully vaccinated.*
[b] Three cubic B-spline basis functions that were used to constitute a non-linear transform for *Age.*

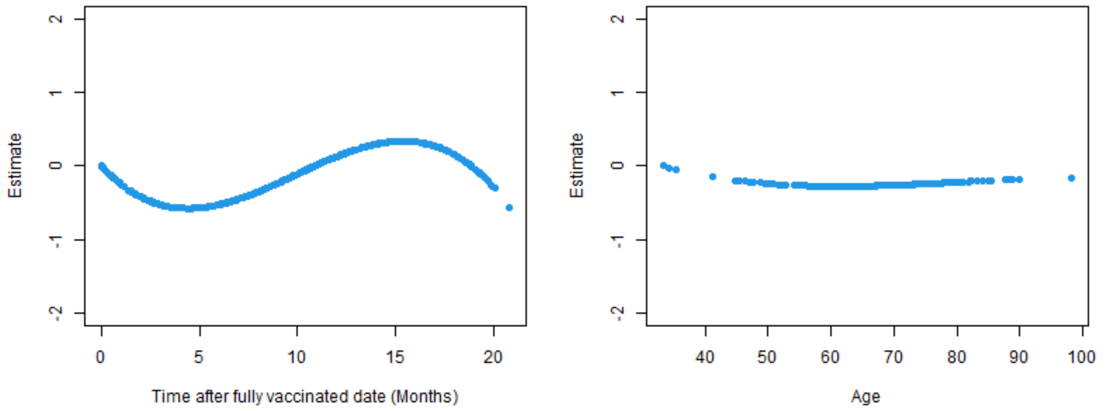

Nonlinear effect of *Time after fully vaccinated* (left) and *Age* (Right) for anti-spike antibody titer.
